# Exosomes derived from human umbilical cord mesenchymal stem cells decrease neuroinflammation and facilitate the restoration of nerve function in rats suffering from intracerebral hemorrhage

**DOI:** 10.1007/s11010-024-04954-w

**Published:** 2024-03-08

**Authors:** Chengrui Nan, Yan Zhang, Aobo Zhang, Yunpeng Shi, Dongdong Yan, Zhimin Sun, Qianxu Jin, Haoran Huo, Yayu Zhuo, Zongmao Zhao

**Affiliations:** 1 Department of Neurosurgery, The Second Hospital of Hebei Medical University, Shijiazhuang, 050000 Hebei China; 2 Department of Neurosurgery, Third Hospital of Shijiazhuang, Shijiazhuang, 050000 Hebei China; 3 Department of Neurosurgery, The Fourth Hospital of Hebei Medical University, Shijiazhuang, 050000 Hebei China

**Keywords:** Intracerebral hemorrhage, Human umbilical cord mesenchymal stem cells, Exosomes, Inflammation, TLR4/NF-κB

## Abstract

Exosomes derived from human umbilical cord mesenchymal stem cells (hUCMSC-ex) have become a hopeful substitute for whole-cell therapy due to their minimal immunogenicity and tumorigenicity. The present study aimed to investigate the hypothesis that hUCMSC-ex can alleviate excessive inflammation resulting from intracerebral hemorrhage (ICH) and facilitate the rehabilitation of the nervous system in rats. In vivo, hemorrhagic stroke was induced by injecting collagenase IV into the striatum of rats using stereotactic techniques. hUCMSC-ex were injected via the tail vein at 6 h after ICH model establishment at a dosage of 200 µg. In vitro, astrocytes were pretreated with hUCMSC-ex and then stimulated with hemin (20 μmol/mL) to establish an ICH cell model. The expression of TLR4/NF-κB signaling pathway proteins and inflammatory factors, including TNF-α, IL-1β, and IL-10, was assessed both in vivo and in vitro to investigate the impact of hUCMSC-ex on inflammation. The neurological function of the ICH rats was evaluated using the corner turn test, forelimb placement test, Longa score, and Bederson score on the 1st, 3rd, and 5th day. Additionally, RT-PCR was employed to examine the mRNA expression of TLR4 following hUCMSC-ex treatment. The findings demonstrated that hUCMSC-ex downregulated the protein expression of TLR4, NF-κB/P65, and p-P65, reduced the levels of pro-inflammatory cytokines TNF-α and IL-1β, and increased the expression of the anti-inflammatory cytokine IL-10. Ultimately, the administration of hUCMSC-ex improved the behavioral performance of the ICH rats. However, the results of PT-PCR indicated that hUCMSC-ex did not affect the expression of TLR4 mRNA induced by ICH, suggesting that hUCMSCs-ex may inhibit TLR4 translation rather than transcription, thereby suppressing the TLR4/NF-κB signaling pathway. We can conclude that hUCMSC-ex mitigates hyperinflammation following ICH by inhibiting the TLR4/NF-κB signaling pathway. This study provides preclinical evidence for the potential future application of hUCMSC-ex in the treatment of cerebral injury.

## Introduction

Nontraumatic intracerebral hemorrhage (ICH) accounts for approximately 15–20% of strokes and is an extremely devastating disease in the brain, leading to a significant number of deaths [1]. The majority of survivors experience significant long-term effects such as impairment in motor skills, thinking abilities, and language [2, 3]. The immediate impact of ICH is the mechanical injury to the brain [4]. However, some patients with ICH showed aggravation despite no hematoma enlargement, suggesting that secondary injury after ICH may be the main cause of this phenomenon [5, 6]. Both clinical and animal studies have demonstrated that this secondary injury involves inflammation, brain swelling, and cell death, ultimately leading to disruption of the blood–brain barrier and extensive loss of brain cells [7–9]. Although the exact molecular mechanisms responsible for secondary damage after ICH are not fully understood, they could potentially serve as promising targets for new therapeutic interventions aimed at preventing further brain injury.

It has been demonstrated that mesenchymal stem cells (MSCs), by virtue of their capacity for differentiation, self-renewal, and immunomodulation, are appropriate for tissue regeneration and the management of diverse diseases as cellular therapies [10–12]. Nevertheless, HUCMSC exhibits certain drawbacks in terms of preserving biological functionality, quantifying bioactive compounds, and ensuring logistical feasibility in clinical treatment [13, 14]. Consequently, the exploration of an alternative cell-free approach with equivalent outcomes and effectiveness appears to be imperative. Researchers have shown interest in exosomes, a type of extracellular vesicle, as a new approach to cell free-based therapies due to their various biological activities and ability to communicate with cells [15, 16]. Evidence suggests that the therapeutic effects of MSCs are achieved through extracellular vesicles (EVs) and the substances they release [17, 18]. Exosomes are small particles ranging in size from 30 to 150 nm and have been found to contain mRNAs, miRNAs, proteins, and growth factors [19, 20]. These contents are considered valuable in targeted therapy, drug delivery, and disease detection. Additionally, exosomes are easily accessible and can cross the blood–brain barrier, making them a convenient option for neurological disorder treatments [21, 22]. Recent findings have confirmed that MSC-Exo is an effective anti-inflammatory agent for neurological injury [23–25]. For example, in a porcine head injury model, BMSC- exosome-treated groups showed lower levels of nuclear factor-κB (NF-κB) compared to controls [26, 27]. In the therapy of subarachnoid hemorrhage in rats, BMSC- exosomes can significantly reduce the levels of inflammatory proteins, such as toll-like receptor 4 (TLR4) and TNF-α [28, 29].

Toll-like receptors (TLRs) are part of a vast group of receptors that detect patterns and are important in the body’s natural defense and inflammatory reactions [30]. TLR4 is triggered by various substances produced within the body, like heme and fibrinogen [31, 32], and its activation leads to a negative outlook after ICH [33, 34]. Once stimulated, the TLR4 signaling pathway is activated and then activates NF-κB. The TLR4/NF-κB pathway regulates transcription of many genes, including pro-inflammatory cytokines and chemokines (such as IL-6), cell cycle genes (such as cyclin D1), anti-apoptotic genes (such as bcl-2), and extracellular proteases (such as MMP3). This pathway results in the p65/p50 heterodimer being released and translocated to the nucleus. The heterodimer then binds to its target, triggering the production and release a variety of inflammatory factors such as IL-6, IL-1β, and TNF-α [35]. These factors play a crucial role in the inflammatory cascade that leads to severe brain damage following ICH. Therefore, a potential treatment strategy for ICH involves inhibiting the translocation of TLR4 signaling from the cytoplasm.

Therefore, we believe that after ICH, the breakdown products of heme and other red blood cells activate the TLR4/NF-κB signaling pathway, thus triggering multiple inflammatory reactions in the nervous system. hUCMSC-ex, by inhibiting the expression of TLR4, blocked the conduction of this signaling pathway and ultimately alleviated excessive inflammatory reactions. Our research will confirm this hypothesis.

## Materials and methods

### Extraction and identification of hUCMSCs

Umbilical cord tissues were purchased from Hebei Benyuan Biotechnology Co. LTD and identified by flow cytometry for surface markers CD73, CD90, and CD105. Human skin fiber cells (hSFCs) were purchased from Sciencell company (Carlsbad, USA).

## Exosome isolation and characterization

Exosomes were obtained by cryogenic ultracentrifugation. First, mesenchymal stem cells were cultured in medium containing 5% CO_2_, 10% FBS without exosomes, and 1% mycillin for 48 h, after which cell supernatants were collected. Second, cells were removed from cell supernatants by centrifugation at 300 g for 10 min. Then, in order to eliminate smaller cellular debris, the supernatants were centrifuged at a speed of 2000 g for a duration of 10 min. And then, the supernatants were centrifuged at a speed of 10,000 g for a duration of 30 min to eliminate apoptotic bodies. Finally, exosomes were collected through ultracentrifugation at a speed of 100,000 g for a duration of 70 min at 4 °C and these isolated exosomes were stored at − 80 °C suspended in PBS. The protein concentration of the exosome was measured by the BCA assay and western blotting was used to assay the exosome representative markers CD63 and CD9 (Abclonal, China). In addition, the morphology and size distribution of exosomes were assessed using transmission electron microscopy (TEM) and nanoparticle tracking analysis (NTA), respectively. The TEM revealed the typical cup-like morphology of hUCMSC-ex and the NTA shows that hUCMSC-ex has a particle size distribution between 30 and 150 nm, with a prominent peak at around 110 nm. The total protein content of exosomes standardized by the BCA assay was 0.5 mg/ml.

### The MSC exosome labeling process.

PKH26 was used to label the exosomes. In short, exosome pellets were suspended again in 1 mL of Diluent C, and then 4 μL of PKH26 was mixed with 1 mL of Diluent C. After combining the exosome suspension with the staining solution, it was left to incubate for a duration of 4 min. To halt the labeling reaction, an equivalent amount of 1% BSA was introduced. After labeling, the exosomes underwent ultracentrifugation at a speed of 100,000 g for a duration of 70 min. Subsequently, they were washed with PBS and subjected to another round of ultracentrifugation.

### Isolation of primary astrocytes

Isoflurane was used to anesthetize newborn rats, and their brains were then removed under aseptic conditions and placed in D-Hank’s solution. Using the anatomical microscope, the cerebellum, brainstem, and hippocampus were extracted, while eliminating the pia and vascular tissues covering the cerebral cortex. Subsequently, the remaining brain tissue was reduced to a dimension of 1 mm^3^. The tiny fragments were submerged in a trypsin solution that contained DNase. The mixture was placed in an incubator set at a temperature of 37 °C for a duration of 5 min. A pipette was used to grind the mixture repeatedly until the majority of the tissue within the mixture has dissolved. The cell suspension was obtained using a cell strainer, centrifuged at 1000 g for 2 min, then the cells were resuspended with cell culture medium and seeded in culture flasks, and the cell culture medium was replaced 24 h later.

### Tracking of MSC exosome administration

PKH26-labeled MSC exosome at a concentration of 10 μg/mL was added to the astrocyte culture medium and incubated for 12 h, followed by immunofluorescence staining to trace MSC-Exo in vitro. The cell samples were stained with Alexa Fluor 488 labeled phalloidin working solution at room temperature for 60 min, away from light. After three rounds of PBS cleaning, the nuclei were stained with sufficient DAPI solution. Images were acquired using a laser confocal microscope.

### Cytotoxicity assay

CCK-8 (Cell Counting Kit-8) was utilized to screen the optimal drug concentration. Astrocytes were cultured in 96-well plates at a density of 5 × 10^3^ cells per well. They were then divided into six groups and subjected to different treatments: PBS, 5, 10, 20, 40, and 60 μmol/mL of hemin for 3 h (*n* = 6). Additionally, another six groups of astrocytes were treated with PBS, hemin, 5, 10, 20, and 50 μg/mL of MSC-Exo for 12 h (*n* = 6). After removing the culture medium, each well was incubated with 10 μl of CCK-8 solution mixed with 100 μl of DMEM for 2 h. Astrocyte viability was assessed by measuring the optical density (OD) of each pore at a wavelength of 450 nm with a microplate reader.

### Cell intracerebral hemorrhage model and siRNA transfection

According to the results of CCK-8 assay, primary astrocytes were exposed to 20 μmol/mL hemin for 3 h to establish a cellular intracerebral hemorrhage model, and the cells were pretreated with exosomes at a concentration of 20 μg/mL for 12 h to ensure the therapeutic effect. In order to transfect siRNA into astrocytes, primary astrocytes from rats were placed in a culture dish containing medium without serum, once the cells reached a fusion level of 60–70%, rat-specific siRNA for TLR4 or nonsense oligonucleotides was introduced using LipofectamineTM 3000 (Invitrogen, L3000015, USA) reagent.

### JC-1 staining

In this study, the JC-1 staining was utilized to measure the mitochondrial membrane potential (MMP) of astrocytes. The cell samples, which consisted of the Control, PBS, hUCMSC-ex, and hSFC-ex groups (*n* = 3), were cultured in 12-well plates. Unlike the other three groups, the control group was not induced by hemin and was treated only with PBS, the hUCMSC-ex group and hSFC-ex group were pretreated with their respective exosomes at a concentration of 10 μg/mL for 12 h, PBS group was treated with PBS replaced. After being washed with PBS three times, the culture was supplemented with 5 mM JC-1 and incubated at 37 °C, 5% CO_2_ for 30 min. Fluorescence microscopy was used to monitor the fluorescence intensity of cells excited at 488 nm (green) and 594 nm (red), and then determined the ratio of green to red fluorescence intensity.

### Immunochemistry

Brain sections were stained for CD68 (Invitrogen, MA5-13324, USA) and MPO (Invitrogen, MA5-16383, USA) using an immunohistochemical kit. In each slide, 5 randomly selected areas were counted for CD68 and MPO-positive inflammatory cells. Cell samples from the Control, PBS, hUCMSC-ex, siRNA, and Nonsense group (*n* = 3). The primary antibodies used were GFAP (1:200) (Abcam, ab7260, China), TLR4 (1: 200) (Abcam, ab13556, China), and mouse NF-κB/P65 (1:200) (Abcam, ab307840, China). After overnight incubation with the primary antibody, the next day samples were incubated with A488 IgG (1:500) (Abcam, ab182931, China) or A594 IgG (1:500) (Abcam, ab150116, China) antibodies for 2 h at room temperature. Cell nuclei were probed with DAPI. Fluorescent microscope (Leica DMi8, Germany) was used to capture images of the cell samples.

### Animal intracerebral hemorrhage model

SD rats used in the experiment were required to be healthy, male, 10–12 weeks old, 200–250 g. Rats were maintained in a controlled temperature (23 ± 1 °C) and humidity (55 ± 2%) environment and were guaranteed free access to food and water. All experimental procedures were approved by the Animal Experiments Ethics Committee of the Second Hospital of Hebei Medical University and strictly followed National Institutes of Health guidelines for animal care and use. A total of 96 SD rats were randomly divided into four groups: Sham, ICH, hUCMSC-ex, and hSFC-ex. Anesthetized rats were fixed in prone position on a stereotactic frame, and the skin was incised along the midline of the skull to expose the anterior fontanelle, posterior fontanelle, and sagittal suture of the skull, and a bone hole was drilled 3 mm to the right of the midline and 0.24 mm posterior to the anterior fontanelle. Then 1 µL collagenase IV was injected into the right striatum at 0.2 µL/min. In sham group, only 1 µL of normal saline was injected without collagenase IV. According to previous studies, the inflammatory response was initiated several hours after ICH, and the activation of M1 microglia peaked at 6 h after ICH. Therefore, exosomes were injected via the tail vein at 6 h after ICH model establishment. Rats in each group received an injection of the same amount of exosomes or normal saline (NS) through the tail vein. The Sham and ICH group received an injection of 200 uL of NS, the hUCMSC-ex group received an injection of 100 µg/200 µL of hUCMSC-ex, and the hSFC-ex group received an injection of 100 µg/200 µL of hSFC-ex.

### Nissl staining

We used toluidine blue Nissl staining to assess neuronal injury. The Nissl staining solution was acquired from Beijing Leagen Biotechnology Co., LTD. Brain tissue sections were deparaffinized with routine xylene and washed with distilled water and then the sections were placed in a solution of toluidine blue stain and incubated at a temperature of 55 °C for a duration of 30 min. After rinsing the sections with distilled water, 70% ethanol, and 95% ethanol for rapid differentiation, and dehydrating them with absolute ethanol, they were finally sealed with neutral gum. Images were collected using a microscope from the perihematomal brain tissue area.

### BBB permeability

The permeability of the blood–brain barrier (BBB) was measured using Evans blue staining. Under anesthesia, the rats were sacrificed after injecting Evan’s Blue Dye (0.5%, 5 mL/kg) slowly into the femoral vein on the right side, one hour prior. Following the infusion of regular chilled saline into the heart, the brain was promptly severed from the body, and the brain matter surrounding the blood clot was measured, blended in 50% TCA (trichloroacetic acid), and spun in a centrifuge. After combining the supernatant with TCA, it was left to incubate at a temperature of 4 °C for the entire night. Following the centrifugation process, the obtained liquid portion was assessed at a wavelength of 620 nm.

### Assessment of brain water content

On day 3 after ICH, the rats were sacrificed after anesthesia and the brain tissue was quickly obtained. The brain tissue was divided into three parts: the left cerebral hemisphere, the right cerebral hemisphere, and the cerebellum. Brain tissue samples were weighed to obtain wet weight (WW) using an electronic analytical balance. To obtain dry weight (DW), brain tissue samples were reweighed after dehydration at 100 °C for 24 h. The brain water content is calculated as computational formula: ${{\left( {{\text{WW}} - {\text{DW}}} \right)} \mathord{\left/ {\vphantom {{\left( {{\text{WW}} - {\text{DW}}} \right)} {{\text{WW*100\%}}}}} \right. \kern-0pt} {{\text{WW*100\% }}}}$.

### Neurobehavioral score

We used the forelimb placement test, the turn corner test, the Bederson score, and the Longa score to assess neurological function in rats after ICH. In the forelimb placement test, when the whiskers of rats were touched to the edge of a table, normal rats were able to quickly place the ipsilateral forelimb on the table, while ICH rats showed impaired motor function of the limb contralateral to the hemorrhage and had difficulty in performing this motion. Each rat was tested 10 times and the number of successes was recorded. For the turn test, the direction choice of the rat was recorded by placing the rat at a 30° angle with a gap enclosed by two walls, and the recording was repeated 10 times to determine the percentage of right turns. The Bederson score consisted of three tests, and the test was terminated when the animal did not perform abnormalities in one of the tests, and the score was the final score. In test 1, the rat’s tail was lifted to 10 cm from the table top and the flexion of its forelimbs was observed. If the rat showed bilateral forelimbs extension, the score was 0 and the test was terminated. If the rat showed one limb flexion and the other limb extension, the score was 1 and the test was entered. In test 2, the rat was pushed from the lateral side and scored two points if the rat showed decreased ability to resist the lateral push, entering test 3. In test 3, the rat was allowed to move freely on the ground and scored three points if the rat exhibited rotation contralateral to the brain injury. In the Longa scale, a score of 0 represented no neurological deficit, a score of 1 indicated that the rat could not fully extend the paralyzed side of the forepaw, a score of 2 indicated that the rat could turn to the paralyzed side while walking, a score of 3 indicated that the rat could not walk automatically, and a score of 4 indicated that the rat could not walk automatically and had a loss of consciousness.

### Western blot

Tissue or cell protein was quantified by BCA method. Proteins from different samples were loaded onto SDS-PAGE and separated by electrophoresis, and then the proteins were transferred to PVDF membranes. Next, nonspecific antigens were blocked with 5% skim milk powder for 1.5 h and then incubated overnight at 4 °C with different dilutions of primary antibodies, including, TLR4 (Abcam, ab13556, 1:200), P65 (Abcam, ab307840, 1:200), p-P65 (Abcam, ab31624, 1:200), and GAPDH (Abclone, AC001, 1:10,000). The following day, PVDF membranes were incubated with fluorescent secondary antibodies (ROCKLAND, 1:10,000) for 2 h at room temperature after repeated rinsing with PBS. Finally, the PVDF membrane was imaged and stored, and the band gray value was measured by ImageJ software. The GAPDH was utilized as an internal standard.

### Enzyme-linked immunosorbent assay (ELISA)

Cell supernatants or animal serum were collected and centrifuged to eliminate any precipitate, and ELISA assay kits (Abclone, RK00020, China) were used to detect the expression levels of TNF-α, IL-1β, and IL-10 in samples.

### RT-PCR

We used an RNA extraction kit to extract total RNA from the samples and measure the concentration of RNA by measuring the absorbance of the RNA solution at 260 nm with a UV spectrophotometer. Then we used the PCR kit to reverse transcribe RNA into cDNA. RT-qPCR was performed according to the instructions provided with SYBR Premix Ex Tap™ II Kit (Takara). GAPDH was used as the internal control for the experimental samples, and the relative gene expression of the samples was calculated using the 2^−ΔΔCt^ method.

### Statistical analysis

We used GraphPad Prism 7 to analyze the data and generate statistical plots, and all summary data are expressed as mean ± SD. Neurological scores were analyzed using the Kruskal–Wallis test and the Dunn multiple comparison test. One-way ANOVA was used for the other values, followed by Tukey’s multiple comparison test. *P* < 0.05 was considered statistically significant.

## Result

### Characterization of hUCMSCs and exosomes

**Fig. 1:**
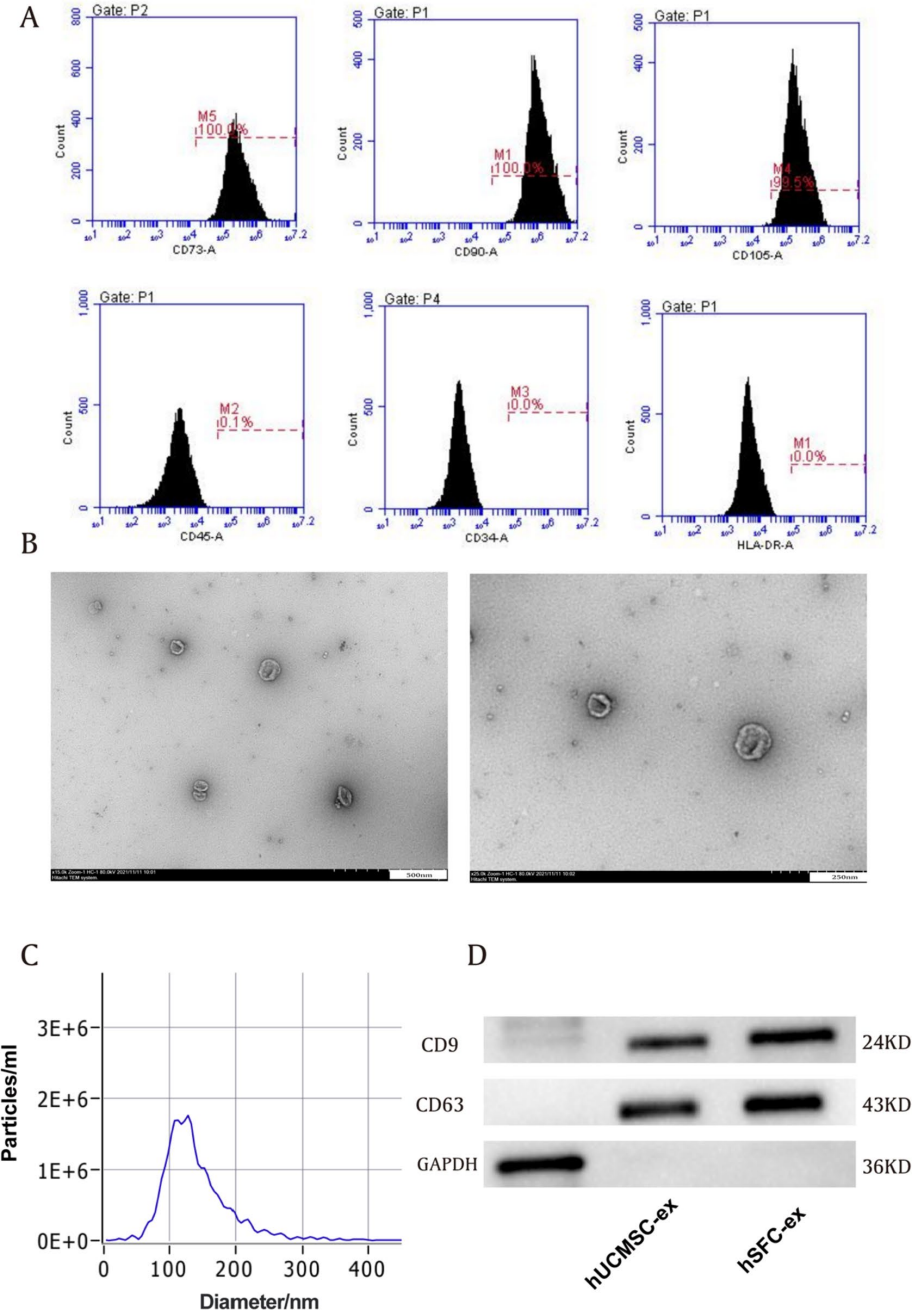
Characterization of hUCMSCs and Exosomes. **A** Flow cytometry analysis showed hUCMSCs express CD73, CD90, and CD105 but not CD34 and CD45. **B** TEM analysis of hUCMSC-ex. Scale bar = 50 nm/100 nm. **C** Dynamic light scattering analysis of hUCMSC-ex. **D** Western blot identification results

Flow cytometry showed that MSCs isolated from human umbilical cord expressed stem cell-specific marker proteins such as CD73, CD90, and CD105, but did not express hematopoietic marker proteins such as CD34 and CD45 (Fig. 1A). To investigate the impact of hUCMSC-ex on the inflammatory response induced by ICH, we obtained exosomes from hUCMSC and hSFC supernatant, hSFC-ex was utilized as a control. Various methods were employed to analyze exosomes. Transmission electron microscopy (TEM) revealed the typical cup-like morphology of hUCMSC-ex (Fig. 1B).

Dynamic light scattering shows that hUCMSC-ex has a particle size distribution between 30 and 150 nm, with a prominent peak at around 110 nm, as illustrated in Fig. 1C. Furthermore, western blot analysis revealed the presence of CD9 and CD63 markers in the exosome samples, indicating their conformity to the standard criteria for exosomes and suitability for future experiments.

### hUCMSC-ex inhibit ICH-induced inflammation and improve MMP in primary astrocytes

**Fig. 2:**
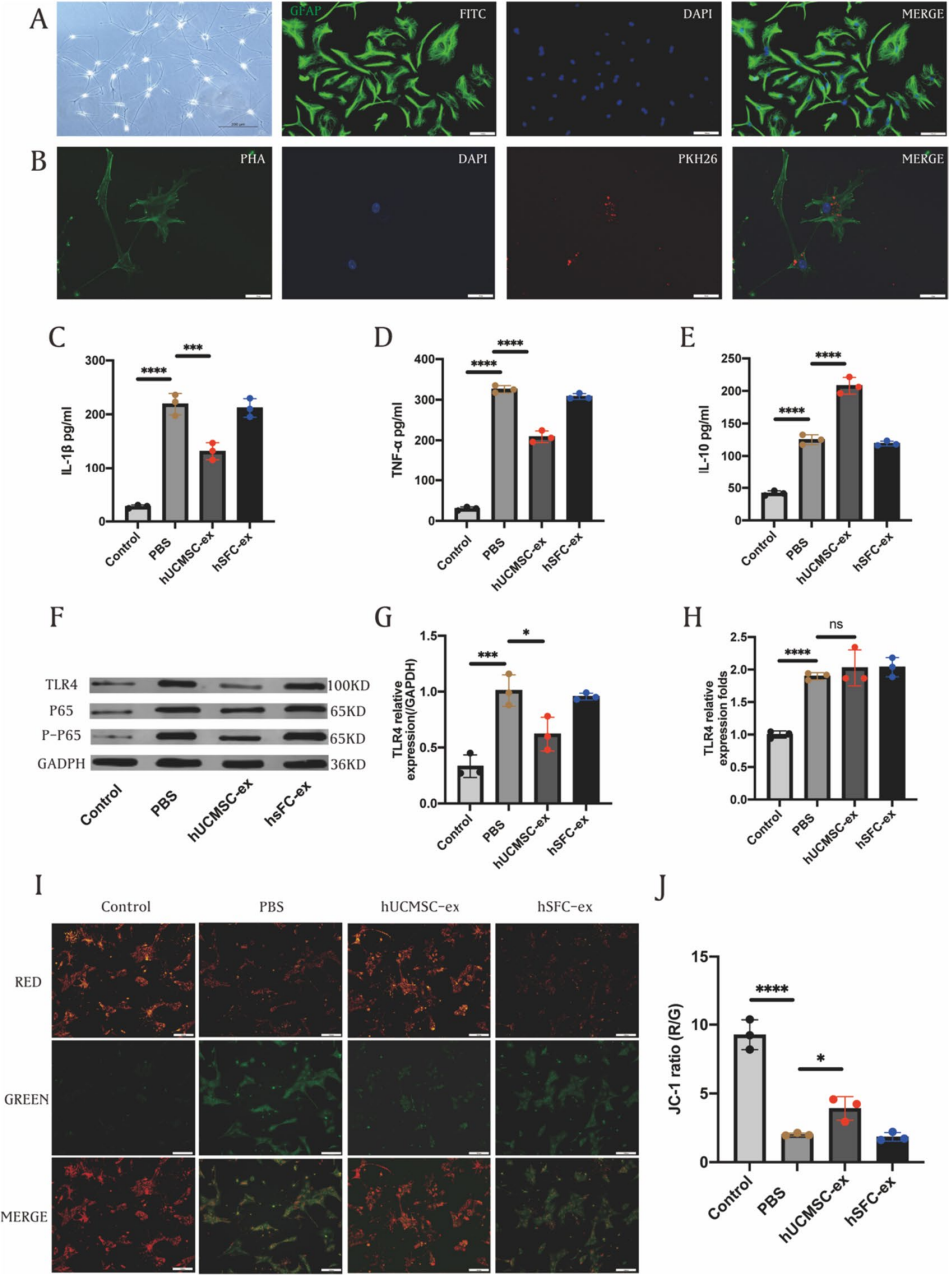
hUCMSC-ex inhibit ICH-induced inflammation and improve MMP in primary astrocytes. **A** Morphological characteristics of Primary astrocytes and their specific marker GFAP. **B** hUCMSC-ex transfected into primary astrocytes. **C–E** ELISA analysis for IL-1β TNF-α, and IL-10 levels in astrocytes supernatants. **F** Western blot for TLR4 and NF-κB/P65, p-P65. **G** Statistical analysis of TLR4. **H** Statistical analysis of TLR4 mRNA. (I) Representative images of the JC-1 staining. **J** The histogram of the ratio of JC-1 fluorescence (Red / Green) in the primary culture of astrocytes. (*n* = 3). Scale bars: A and H, 50 μm; B, 100 μm. Data were expressed as mean ± standard deviation. *p < 0.05, **p < 0.01, ***p < 0.001, ****p < 0.0001

In order to determine the mechanism by which hUCMSC-ex treats ICH, we utilized primary astrocytes that were stimulated by hemin as a cell model for ICH. Immunofluorescence staining was employed to analyze the cultured cells, and the findings indicated that GFAP, a distinctive astrocyte marker, was expressed by all cells (Fig. 2A). To track hUCMSC-ex in vitro, we transfected primary rat astrocytes with PKH26-labeled exosomes and stained astrocytes with phalloidin. We found that red-labeled hUCMSC-ex nanoparticles were distributed in the cytoplasm or processes (Fig. 2B). The ELISA results showed that ICH stimulation observably increased the levels of IL-1β (Fig. 2C) and TNF-a (Fig. 2D) in the supernatant of astrocytes, while hUCMSC-ex treatment effectively reduced their levels. Meanwhile, IL-10 (Fig. 2E) showed an opposite expression trend. The Western blot findings demonstrated that TLR4, NF- κB/P65, and p-P65 protein levels were observably elevated in astrocytes intervened by hemin, in comparison to the control group. However, the administration of hUCMSC-ex efficaciously reduced the expression of these proteins, as depicted in Fig. 2F. hSFC-ex did not have any impact on the levels of inflammatory proteins following ICH stimulation, in contrast to hUCMSC-ex. The quantitative data also indicated that the intervention of hUCMSC-ex resulted in a noteworthy decrease in the protein level of TLR4 (Fig. 2G). According to RT-PCR analysis, it was observed that the levels of TLR4 mRNA increased following ICH stimulation in comparison to the control group. However, the administration of hUCMSC-ex did not have any impact on the expression of TLR4 mRNA, as depicted in Fig. 2H. To assess the impact of hUCMSC-ex on cell survival, the MMP of astrocytes was evaluated by utilizing the mitochondria-specific dye JC-1. The statistical analysis showed a notable decrease in the JC-1 ratio within the ICH group, which was enhanced by the administration of hUCMSC-ex (Fig. 2I-J). Collectively, this information indicates that the administration of hUCMSC-ex improved the inflammatory response and mitochondrial impairment in astrocytes caused by ICH.

### hUCMSC-ex can reduce the brain injury caused by ICH

**Fig. 3:**
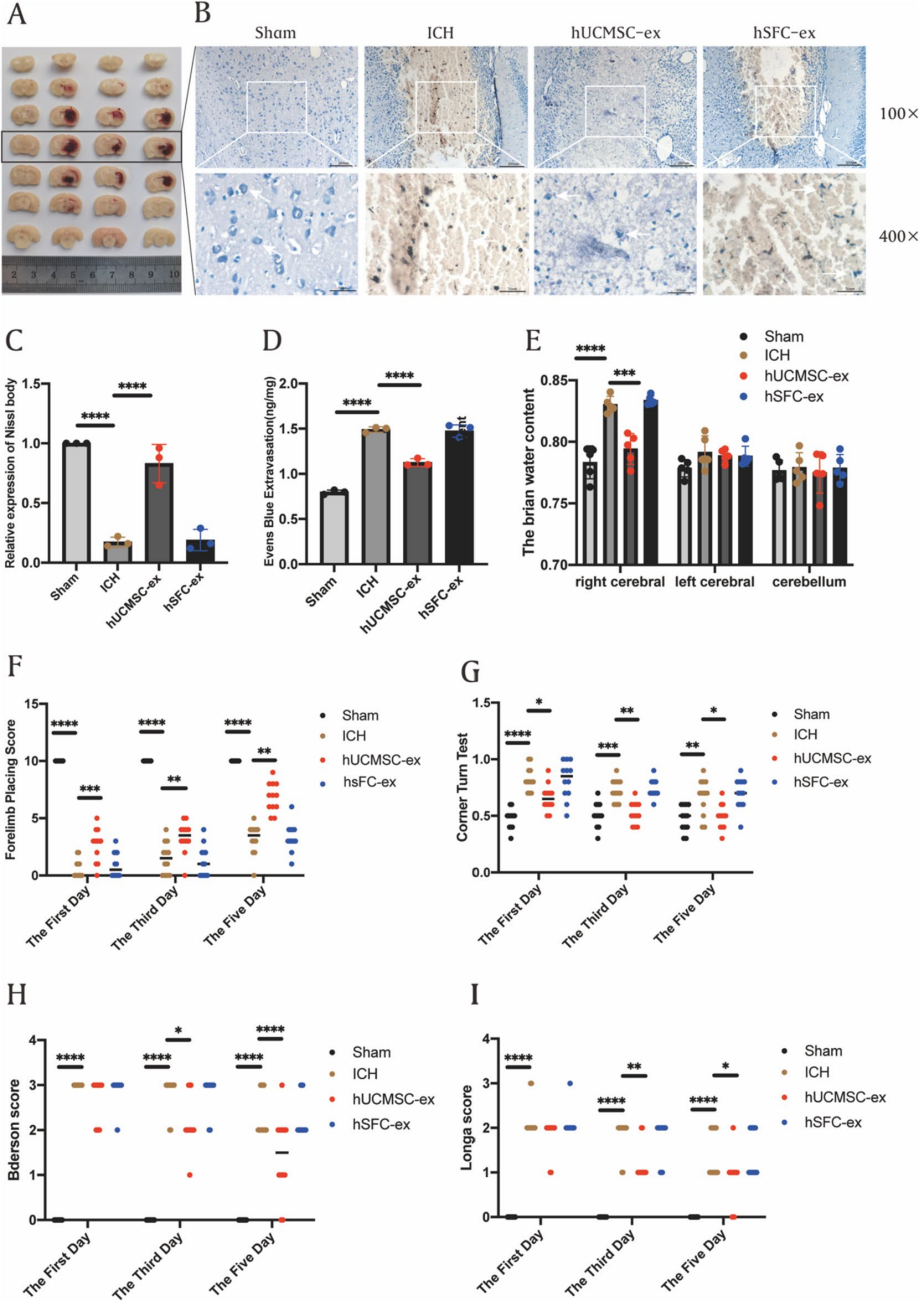
hUCMSC-ex reduce the brain injury caused by ICH. **A** Representative pictures of the hemorrhagic lesion. **B** Nissl staining in the Rat of different groups. **C** Representative pictures of the Evens blue extravasation of different groups. (*n* = 3). **D** The cerebral water content of each group was examined to evaluate the effect of treatment on cerebral edema. (*n* = 5). **E** The forelimb placement test, (*n* = 10). **F** The corner turn test. (*n* = 10). The **G** Longa and **H** Pedersen scores were performed to evaluate from day 1 to day 5 after ICH to assess recovery of neural function. (*n* = 10). Data were expressed as mean ± standard deviation. *p < 0.05, **p < 0.01, ***p < 0.001, ****p < 0.0001

As shown in brain tissue sections, treatment with hUCMSC-ex significantly reduced hematoma volume compared with the control and hSFC-ex-treated groups (Fig. 3A). Compared to the control group, the ICH group and hSFC-ex group exhibited evident cell necrosis and a notable decrease in Nissl bodies in the brain tissue surrounding the hemorrhage, as demonstrated by Nissl staining (Fig. 3B). The hUCMSC-ex group exhibited a notable decrease in pathological harm and a rise in the quantity of Nissl bodies, as shown in Fig. 3C. The permeability of the blood–brain barrier was evaluated using the Evans blue leakage technique, revealing an observably increase in extravasation in the ICH group compared to the sham group. Additionally, the administration of hUCMSC-ex improved the worsening of extravasation, as depicted in Fig. 3D. On the fifth day following ICH, the cerebral edema therapeutic effect was assessed by measuring the brain’s water content. The findings indicated a notable decrease in brain water content in the hUCMSC-ex group (Fig. 3E). Neurobehavioral assessments were conducted on rats with ICH at 1, 3, and 5 days after the occurrence to identify the impact of hUCMSC-ex on neurological impairments. To quantitatively assess sensorimotor function, the forelimb placement test and corner turn test were employed, while neurological function was evaluated using the Longa and Bedersen scores. Compared to the ICH group, hUCMSC-ex greatly enhanced the accurate placement of the forelimb in ICH rats (Fig. 3F) and have achieved better results in the corner turn test (Fig. 3G). The ICH group experienced a significant decrease in neurological function score 24 h after the operation. However, neurological function showed significant improvement following treatment with hUCMSC-ex (Fig. 3H-I).

### hUCMSC-ex alleviated the inflammatory response of rats after ICH

**Fig. 4:**
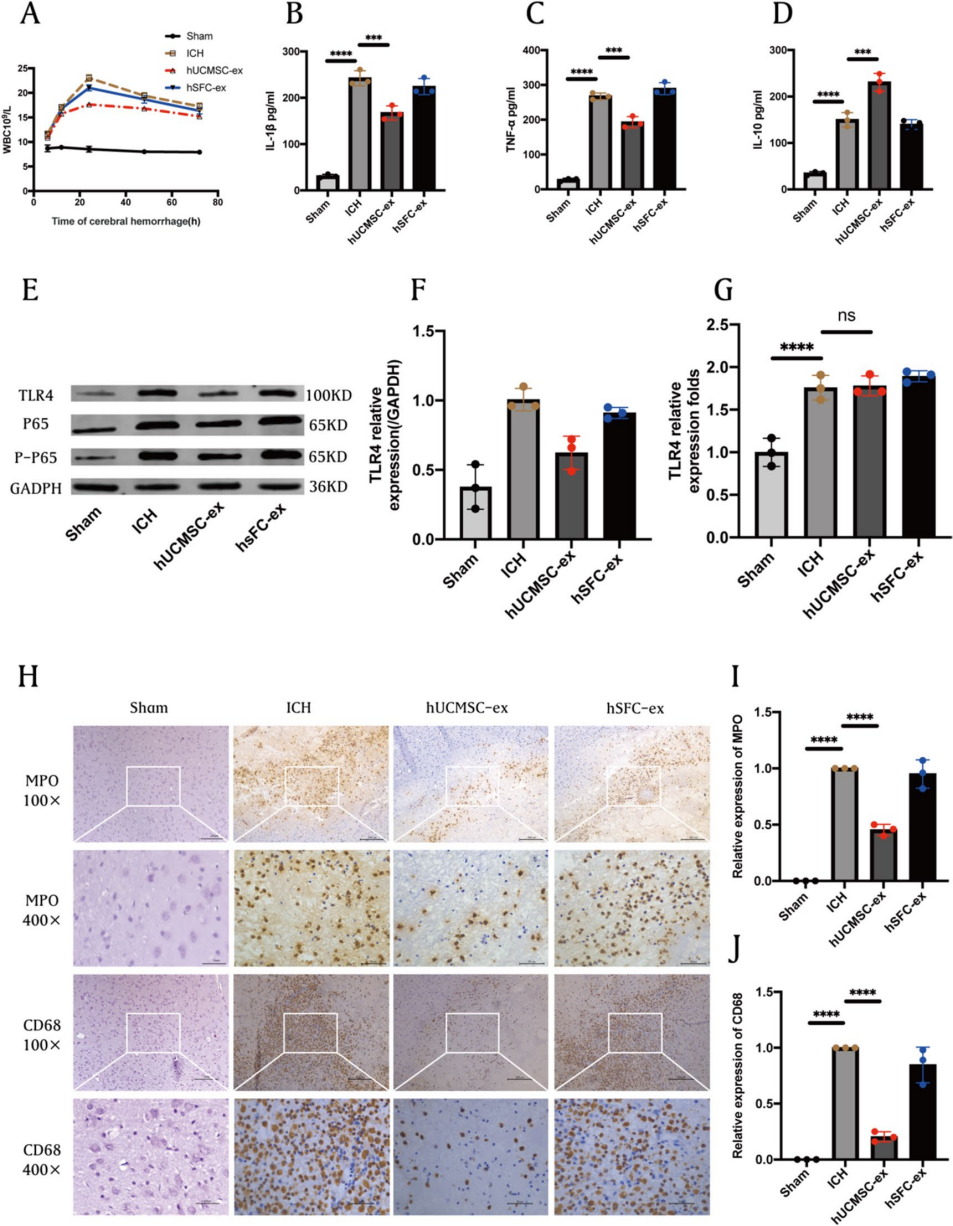
hUCMSC-ex alleviated the inflammatory response of rats after ICH. **A** Total WBC in different groups. **B–D** The concentrations of IL-1β, TNF-α, and IL-10 in serum were detected by ELISA. **E** Western blot for TLR4 and NF-κB/P65, p-P65. **F** Statistical analysis of TLR4. **G** Statistical analysis of TLR4 mRNA. **H** Representative immunohistochemical staining of MPO and CD68 in different groups and **I** and **J** statistical analysis (*n* = 3). Scale bars: 200 μm (100 ×), 50 μm (400 ×). Data were expressed as mean ± standard deviation. *p < 0.05, **p < 0.01, ***p < 0.001, ****p < 0.0001

We measured the total number of white blood cells in the peripheral blood of rats after ICH. The results showed that ICH could significantly increase the number of white blood cells, but hUCMSC-ex treatment could significantly inhibit the increase, particularly at 24 h after ICH, while hSFC-ex had no such result (Fig. 4A). The ELISA results showed that the expression of pro-inflammatory factors IL-1β (Fig. 4B) and TNF-α (Fig. 4C) in the serum of rats after ICH was increased, but the anti-inflammatory factor IL-10 (Fig. 4D) was decreased. However, hUCMSC-ex treatment could improve this trend. Western blot results showed that hUCMSC-ex administration observably reduced the expression of TLR4, NF-κB/P65 and p-P65 proteins (Fig. 4E-F). The RT-PCR analysis revealed an elevation in TLR4 mRNA levels following ICH stimulation compared with control group. However, the administration of hUCMSC-ex did not impact the expression of TLR4 mRNA. Immunohistochemical staining showed that neutrophil (MPO) and macrophage (CD68) counts were significantly increased in perihematomal brain tissue of ICH rats. However, hUCMSC-ex treatment significantly decreased their number (Fig. 4G). Additionally, quantitative analysis was conducted utilizing the Image J software (Fig. 4I-J). In general, the administration of hUCMSC-ex effectively reduced inflammation following ICH.

### hUCMSC-ex inhibit ICH-induced hyperinflammation by targeting TLR4/NF-κB signaling pathway

**Fig. 5:**
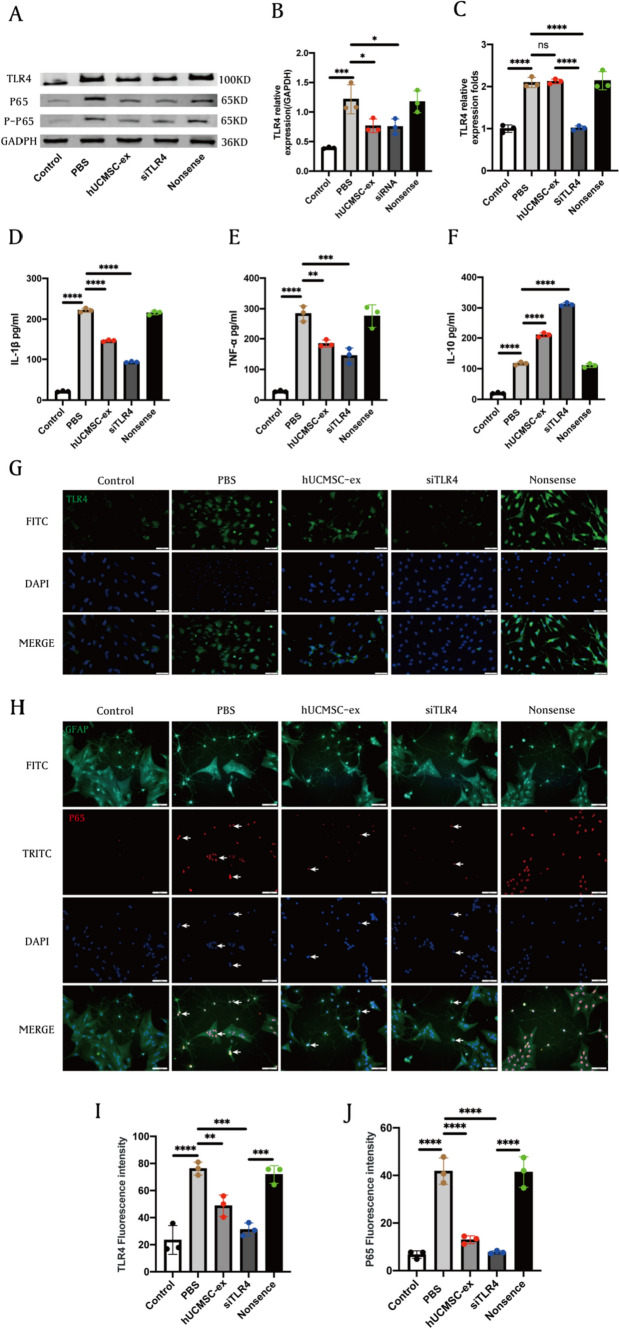
hUCMSC-ex inhibit ICH-induced hyperinflammation by targeting TLR4/NF-κB signaling pathway. **A** Western blot for TLR4 and NF-κB/P65, p-P65. **B** Statistical analysis of TLR4. **C** Statistical analysis of TLR4 mRNA. **D**–**F** ELISA analysis for IL-1β TNF-α, and IL-10 levels in different groups. **G** Representative immunofluorescence images of TLR4 expression in astrocytes and **I** statistical analysis. **H** Representative images of the nuclear translocation of P65 (white arrows) in ICH-induced astrocytes and **J** statistical analysis. (*n* = 3). Scale bars: **G** 50 μm; **H** 100 μm. Data were expressed as mean ± standard deviation. *p < 0.05, **p < 0.01, ***p < 0.001, ****p < 0.0001

The TLR4 signaling pathway serves as a crucial regulator of the inflammatory response. Our experiments also validated that hUCMSC-ex suppressed the expression of TLR4 and its downstream target proteins NF-κB/P65 and p-P65, both in vitro and in vivo. To investigate the mechanism through which hUCMSC-ex inhibits inflammatory reactions, primary astrocytes were transfected with TLR4-specific siRNA or nonsense fragments, and then treated with hemin after 3 h to establish an ICH model. The western blot results revealed a significant decrease in the expression of TLR4, NF-κB/P65, and p-P65 proteins in the hUCMSC-ex group and TLR4 siRNA group at 24 h compared to the PBS group (Fig. 5A-B). RT-PCR analysis revealed that the administration of hUCMSC-ex had no impact on the expression of TLR4 mRNA, but the TLR4 siRNA transfection group was significantly reduced (Fig. 5C). Similarly, ELISA results showed that hUCMSC-ex treatment or TLR4 siRNA significantly reduced IL-1β (Fig. 5D) and TNF-a (Fig. 5E) levels released by astrocytes, while increasing IL-10 (Fig. 5F) expression levels. We observed the effects of hUCMSC-ex and siRNA on TLR4 expression in astrocytes by immunofluorescence staining. Our findings revealed a significant increase in TLR4 expression after ICH exposure, whereas administration of hUCMSC-ex or transfection of TLR4 siRNA notably decreased TLR4 expression (Fig. 5G-I). Immunofluorescence was employed to further investigate the movement of inflammatory factors into the nucleus. Our findings indicated that the nuclear translocation of P65 in cultured astrocytes in response to ICH stimulation was hindered by the administration of hUCMSC-ex treatment and TLR4 siRNA, as demonstrated in Fig. 5H and Fig. 5J. The findings indicated that hUCMSC-ex suppressed inflammation caused by ICH by reducing the activity of the TLR4/NF-κB signaling pathway.

## Discussion

Our study aimed to explore the potential protective impact of hUCMSC-ex on cerebral injury caused by ICH and its effects. The key discoveries can be summarized as follows: [1] A solitary systematic delivery of 200 µg hUCMSC-ex resulted in a significant decrease in hematoma volume, alleviation of brain edema, enhancement of the blood–brain barrier, and improvement in behavioral performance among ICH rats. [2] In vitro, hUCMSC-ex suppressed the inflammatory reaction of astrocytes triggered by hemin and enhanced the potential of mitochondrial membrane. In vivo, hUCMSC-ex also exhibited suppressive properties against neuroinflammation induced by ICH. [3] ICH triggered the activation of the TLR4/NF-κB pathway, while hUCMSC-ex effectively suppressed the neuroinflammatory response in the brain by targeting the TLR4/NF-κB pathway.

There is growing evidence that inflammatory responses play a crucial role in secondary brain injury, and the use of anti-inflammatory treatments in intracerebral hemorrhage may be very important [36–38]. Activation of microglia and astrocytes occurs within minutes following ICH [39, 40], and this activation can result in the release of various cytokines, free radicals, and other potentially harmful substances, leading to secondary brain injury caused by ICH [41]. In addition to microglia, other inflammatory cells from the blood, such as neutrophils and macrophages, are also activated and involved in brain damage caused by ICH [42]. The activation of NF-κB is a widely recognized outcome of TLR signaling pathways, leading to the synthesis of pro-inflammatory cytokines [43]. Accumulating evidence suggests that the TLR signaling pathway plays an important role in the inflammatory response caused by brain injury [44]. TLR is a group of transmembrane proteins which can recognize and bind various ligands through the extracellular domain, resulting in conformational changes to the receptor, which then recruit intracellular adaptor proteins, including MyD88, TRIF, and TIRAP. The recruited adaptor protein initiates downstream signaling events that ultimately lead to the expression of NF-κB and the inflammatory agents IL-6, TNF-α, and IL-1β. The expression of TLR4 is significantly increased with the infiltration of inflammatory cells around the hematoma within a few hours after intracerebral hemorrhage [45–47]. Our study also confirmed that ICH can activate the TLR4/NF-κB signaling pathway, leading to infiltration of neutrophils and macrophages and increased expression of inflammatory factors. Therefore, targeting TLR4 and its signaling pathway may be an effective method to treat ICH.

Mesenchymal stem cells (MSCs) have attracted great attention as a kind of cell therapy to treat diseases due to their multifaceted differentiation, self-renewal, and immune response capabilities [48]. Mesenchymal stem cells can be derived from a variety of tissues in the human body [49]. Among these sources, hUCMSCs become an ideal therapeutic method due to their advantages of easy extraction and expansion and low immunogenicity [50]. Many studies have demonstrated the potential of hUCMSCs to treat diseases such as diabetes [51], brain diseases [52], and cardiovascular diseases [53]. However, there are still challenges associated with the direct application of stem cells, such as potential side effects like tumorigenicity, pulmonary embolism, and insufficient stem cell migration across the blood–brain barrier [54]. Exosomes, which are small vesicles with a diameter ranging from 30 to 150 nm, can be obtained through centrifugation at 100,000 g and preserved at -80 °C without affecting their biological activity [55]. The compact size of exosomes enables them to easily traverse the blood–brain barrier. In addition, they carry a large number of bioactive substances with anti-inflammatory effects, such as mRNA, miRNA, and growth factors, and exhibit lower immunogenicity and are easier to store and treat than MSCs [56].

In our research, we isolated exosomes from hUCMSCs and we used TEM and Western Blot to analyze their surface characteristics, which confirmed the presence of specific proteins like CD63 and CD9. Subsequently, we incubated exosomes labeled with PKH26 with hUCMSCs and observed that these exosomes can merge with primary astrocytes in vitro and this finding has important implications for the future development of exosomes as targeted drugs. To examine the impact of hUCMSC-ex on astrocytes, we first treated astrocytes with these exosomes and then stimulated them with hemin to create a cell ICH model. The expressions of TLR4, NF-κB/P65, and p-P65 in Hemin-stimulated astrocytes were significantly increased, along with the increased expressions of pro-inflammatory factors TNF-α and IL-1β in cell supernatant and the decreased expression of IL-10 in anti-inflammatory cells. However, the use of hUCMSC-ex significantly ameliorates these changes. To assess the effect of hUCMSC-ex on MMP in primary astrocytes, we used JC-1 staining which revealed a notable decrease in the JC-1 ratio in Hemin-stimulated primary astrocytes, but was improved by treatment with hUCMSC-ex. In vivo, hUCMSC-ex has a significant inhibitory effect on excessive inflammation caused by ICH, reducing the number of inflammatory cells, down-regulating the expression of TLR4, NF-κB/P65 and p-P65 proteins, thereby preventing the release of inflammatory factors such as IL-1β and TNF-α.

Additionally, hUCMSC-ex decreased the infiltration of neutrophils and macrophages in the brain tissue surrounding the hematoma, alleviated brain edema, and improved the integrity of the blood–brain barrier. Thus, we hypothesized that the anti-inflammatory action of hUCMSC-ex could be linked to the inhibition of the TLR4/NF-κB signaling pathway. Intriguingly, both in vivo and in vitro RT-PCR examinations revealed elevated levels of TLR4 mRNA following ICH stimulation in comparison to the control group, while hUCMSC-ex did not exert any influence on TLR4 mRNA expression. To gain further insight into the anti-inflammatory mechanism of hUCMSC-ex, we employed siRNA to silence TLR4 gene expression. The findings indicated that, in comparison to hUCMSC-ex, the siTLR4 group exhibited reduced protein levels of TLR4 and NF-κB, as well as decreased nuclear translocation of P65. Considering the previous RT-PCR outcomes, our hypothesis is that hUCMSC-ex potentially suppresses TLR4 translation instead of transcription, consequently inhibiting the TLR4/NF-κB signaling pathway. Undoubtedly, the functioning of hUCMSC-ex is exceedingly intricate, necessitating further extensive research in the forthcoming times.

There are two major limitations in this study that could be addressed in future research. First, the study focused on the effect of hUCMSC-ex on excessive inflammation caused by ICH, which in fact is only one type of secondary brain injury caused by ICH. Second, the dose and mode of administration of hUCMSC-ex in the rat ICH model are relatively simple, which may affect the judgment of the therapeutic effect of hUCMSC-ex. In the following studies, we will further analyze the effects of hUCMSC-ex on apoptosis and oxidative stress induced by ICH, and test the effects of different doses and routes of administration on the neural function of ICH rats.

Through our study, we concluded that the breakdown products of blood cells after ICH as endogenous ligands activate the TLR4/NF-κB inflammatory signaling pathway, leading to the large expression of NF-κB and inflammatory cytokines IL-1β and TNF-α, and ultimately lead to excessive inflammation of the nervous system. hUCMSC-ex blocks this signaling pathway by affecting the expression of TLR4 protein. We speculate that the molecular mechanism of hUCMSC-ex may be that some miRNAs in exosomes inhibit the expression of TLR4 protein. In animal experiments, we also found that hUCMSC-ex can not only reduce the inflammatory response induced by ICH, but also reduce brain edema, improve blood–brain barrier function, and ultimately improve the neurological function of ICH rats. Patients with ICH will show increased body temperature, increased white blood cells, high CRP (c-reactive protein) and other inflammatory reactions, as well as increased cerebral edema and destruction of blood–brain barrier, etc. Our findings provide evidence for the future clinical application of hUCMSC-ex in ICH. In future clinical trials, we will try to use hUCMSC-ex to treat patients with ICH by intravenous or intrathecal injection, and evaluate the changes in patients’ inflammatory response and sensorimotor function.

## Publisher’s note

All claims expressed in this article are solely those of the authors and do not necessarily represent those of their affiliated organizations, or those of the publisher, the editors, and the reviewers. Any product that may be evaluated in this article, or claim that may be made by its manufacturer, is not guaranteed or endorsed by the publisher.

## Data Availability

The data used to support the findings of this study are included in the article.

## References

[1] Feigin VL, Krishnamurthi RV, Parmar P, Norrving B, Mensah GA, Bennett DA et al (2015) Update on the global burden of ischemic and hemorrhagic stroke in 1990–2013: the GBD 2013 study. Neuroepidemiology 45(3):161–176 26505981 10.1159/000441085 PMC4633282

[2] Chen G, Leak RK, Sun Q, Zhang JH, Chen J (2018) Neurobiology of stroke: research progress and perspectives. Prog Neurobiol 163–164:1–4 29733886 10.1016/j.pneurobio.2018.05.003

[3] Wilkinson DA, Pandey AS, Thompson BG, Keep RF, Hua Y, Xi G (2018) Injury mechanisms in acute intracerebral hemorrhage. Neuropharmacology 134(Pt B):240–248 28947377 10.1016/j.neuropharm.2017.09.033 PMC6027647

[4] Brouwers HB, Goldstein JN (2012) Therapeutic strategies in acute intracerebral hemorrhage. Neurotherapeutics 9(1):87–98 22139592 10.1007/s13311-011-0091-8 PMC3271150

[5] Babu R, Bagley JH, Di C, Friedman AH, Adamson C (2012) Thrombin and hemin as central factors in the mechanisms of intracerebral hemorrhage-induced secondary brain injury and as potential targets for intervention. Neurosurg Focus 32(4):E8 22463118 10.3171/2012.1.FOCUS11366

[6] Elliott J, Smith M (2010) The acute management of intracerebral hemorrhage: a clinical review. Anesth Analg 110(5):1419–1427 20332192 10.1213/ANE.0b013e3181d568c8

[7] Lee MJ, Cha J, Choi HA, Woo SY, Kim S, Wang SJ et al (2017) Blood-brain barrier breakdown in reversible cerebral vasoconstriction syndrome: Implications for pathophysiology and diagnosis. Ann Neurol 81(3):454–466 28195428 10.1002/ana.24891

[8] Lan X, Han X, Liu X, Wang J (2019) Inflammatory responses after intracerebral hemorrhage: from cellular function to therapeutic targets. J Cereb Blood Flow Metab 39(1):184–186 30346222 10.1177/0271678X18805675 PMC6311675

[9] Selim M, Norton C (2020) Perihematomal edema: implications for intracerebral hemorrhage research and therapeutic advances. J Neurosci Res 98(1):212–218 30575082 10.1002/jnr.24372 PMC6588515

[10] Ren H, Sang Y, Zhang F, Liu Z, Qi N, Chen Y (2016) Comparative analysis of human mesenchymal stem cells from umbilical cord, dental pulp, and menstrual blood as sources for cell therapy. Stem Cells Int 2016:3516574 26880954 10.1155/2016/3516574 PMC4736971

[11] Castro-Manrreza ME, Montesinos JJ (2015) Immunoregulation by mesenchymal stem cells: biological aspects and clinical applications. J Immunol Res 2015:394917 25961059 10.1155/2015/394917 PMC4417567

[12] Abbaszadeh H, Ghorbani F, Derakhshani M, Movassaghpour A, Yousefi M (2020) Human umbilical cord mesenchymal stem cell-derived extracellular vesicles: a novel therapeutic paradigm. J Cell Physiol 235(2):706–717 31254289 10.1002/jcp.29004

[13] Ding M, Shen Y, Wang P, Xie Z, Xu S, Zhu Z et al (2018) Exosomes isolated from human umbilical cord mesenchymal stem cells alleviate neuroinflammation and reduce amyloid-beta deposition by modulating microglial activation in Alzheimer’s disease. Neurochem Res 43(11):2165–2177 30259257 10.1007/s11064-018-2641-5

[14] Kumar A, Rajendran V, Sethumadhavan R, Purohit R (2012) In silico prediction of a disease-associated STIL mutant and its affect on the recruitment of centromere protein J (CENPJ). FEBS Open Bio 2:285–293 23772360 10.1016/j.fob.2012.09.003 PMC3678130

[15] Zhou JF, Xiong Y, Kang X, Pan Z, Zhu Q, Goldbrunner R et al (2022) Application of stem cells and exosomes in the treatment of intracerebral hemorrhage: an update. Stem Cell Res Ther 13(1):281 35765072 10.1186/s13287-022-02965-2 PMC9241288

[16] Zhang W, Wang Y, Kong J, Dong M, Duan H, Chen S (2017) Therapeutic efficacy of neural stem cells originating from umbilical cord-derived mesenchymal stem cells in diabetic retinopathy. Sci Rep 7(1):408 28341839 10.1038/s41598-017-00298-2 PMC5412648

[17] Shi Y, Wang Y, Li Q, Liu K, Hou J, Shao C et al (2018) Immunoregulatory mechanisms of mesenchymal stem and stromal cells in inflammatory diseases. Nat Rev Nephrol 14(8):493–507 29895977 10.1038/s41581-018-0023-5

[18] Tanwar G, Mazumder AG, Bhardwaj V, Kumari S, Bharti R, Yamini et al (2019) Target identification, screening and in vivo evaluation of pyrrolone-fused benzosuberene compounds against human epilepsy using Zebrafish model of pentylenetetrazol-induced seizures. Sci Rep. 9(1):7904 31133639 10.1038/s41598-019-44264-6 PMC6536720

[19] El Andaloussi S, Mäger I, Breakefield XO, Wood MJ (2013) Extracellular vesicles: biology and emerging therapeutic opportunities. Nat Rev Drug Discov. 12(5):347–57 23584393 10.1038/nrd3978

[20] Phinney DG, Pittenger MF (2017) Concise review: MSC-derived exosomes for cell-free therapy. Stem Cells 35(4):851–858 28294454 10.1002/stem.2575

[21] Long Q, Upadhya D, Hattiangady B, Kim DK, An SY, Shuai B et al (2017) Intranasal MSC-derived A1-exosomes ease inflammation, and prevent abnormal neurogenesis and memory dysfunction after status epilepticus. Proc Natl Acad Sci USA 114(17):E3536–E3545 28396435 10.1073/pnas.1703920114 PMC5410779

[22] Bhardwaj V, Purohit R (2020) Computational investigation on effect of mutations in PCNA resulting in structural perturbations and inhibition of mismatch repair pathway. J Biomol Struct Dyn 38(7):1963–1974 31138032 10.1080/07391102.2019.1621210

[23] Drommelschmidt K, Serdar M, Bendix I, Herz J, Bertling F, Prager S et al (2017) Mesenchymal stem cell-derived extracellular vesicles ameliorate inflammation-induced preterm brain injury. Brain Behav Immun 60:220–232 27847282 10.1016/j.bbi.2016.11.011

[24] Yang Y, Ye Y, Su X, He J, Bai W, He X (2017) MSCs-derived exosomes and neuroinflammation, neurogenesis and therapy of traumatic brain injury. Front Cell Neurosci 11:55 28293177 10.3389/fncel.2017.00055 PMC5329010

[25] Xian P, Hei Y, Wang R, Wang T, Yang J, Li J et al (2019) Mesenchymal stem cell-derived exosomes as a nanotherapeutic agent for amelioration of inflammation-induced astrocyte alterations in mice. Theranostics 9(20):5956–5975 31534531 10.7150/thno.33872 PMC6735367

[26] Williams AM, Wu Z, Bhatti UF, Biesterveld BE, Kemp MT, Wakam GK et al (2020) Early single-dose exosome treatment improves neurologic outcomes in a 7-day swine model of traumatic brain injury and hemorrhagic shock. J Trauma Acute Care Surg 89(2):388–396 32218019 10.1097/TA.0000000000002698

[27] Singh R, Bhardwaj V, Purohit R (2021) Identification of a novel binding mechanism of Quinoline based molecules with lactate dehydrogenase of Plasmodium falciparum. J Biomol Struct Dyn 39(1):348–356 31903852 10.1080/07391102.2020.1711809

[28] Xiong L, Sun L, Zhang Y, Peng J, Yan J, Liu X (2020) Exosomes from bone marrow mesenchymal stem cells can alleviate early brain injury after subarachnoid hemorrhage through miRNA129-5p-HMGB1 pathway. Stem Cells Dev 29(4):212–221 31801411 10.1089/scd.2019.0206

[29] Kumar Bhardwaj V, Purohit R, Kumar S (2021) Himalayan bioactive molecules as potential entry inhibitors for the human immunodeficiency virus. Food Chem 347:128932 33465692 10.1016/j.foodchem.2020.128932

[30] Kong Y, Le Y (2011) Toll-like receptors in inflammation of the central nervous system. Int Immunopharmacol 11(10):1407–1414 21600311 10.1016/j.intimp.2011.04.025

[31] Figueiredo RT, Fernandez PL, Mourao-Sa DS, Porto BN, Dutra FF, Alves LS et al (2007) Characterization of heme as activator of Toll-like receptor 4. J Biol Chem 282(28):20221–20229 17502383 10.1074/jbc.M610737200

[32] Smiley ST, King JA, Hancock WW (2001) Fibrinogen stimulates macrophage chemokine secretion through toll-like receptor 4. J Immunol 167(5):2887–2894 11509636 10.4049/jimmunol.167.5.2887

[33] Sansing LH, Harris TH, Welsh FA, Kasner SE, Hunter CA, Kariko K (2011) Toll-like receptor 4 contributes to poor outcome after intracerebral hemorrhage. Ann Neurol 70(4):646–656 22028224 10.1002/ana.22528 PMC3671585

[34] Singh R, Bhardwaj VK, Purohit R (2022) Computational targeting of allosteric site of MEK1 by quinoline-based molecules. Cell Biochem Funct 40(5):481–490 35604288 10.1002/cbf.3709

[35] Yu L, Chen C, Wang LF, Kuang X, Liu K, Zhang H et al (2013) Neuroprotective effect of kaempferol glycosides against brain injury and neuroinflammation by inhibiting the activation of NF-κB and STAT3 in transient focal stroke. PLoS ONE 8(2):e55839 23437066 10.1371/journal.pone.0055839 PMC3577792

[36] Jin R, Yang G, Li G (2010) Inflammatory mechanisms in ischemic stroke: role of inflammatory cells. J Leukoc Biol 87(5):779–789 20130219 10.1189/jlb.1109766 PMC2858674

[37] Maida CD, Norrito RL, Daidone M, Tuttolomondo A, Pinto A (2020) Neuroinflammatory mechanisms in ischemic stroke: focus on cardioembolic stroke, background, and therapeutic approaches. Int J Mol Sci. 21(18):6454 32899616 10.3390/ijms21186454 PMC7555650

[38] Ahmad M, Dar NJ, Bhat ZS, Hussain A, Shah A, Liu H et al (2014) Inflammation in ischemic stroke: mechanisms, consequences and possible drug targets. CNS Neurol Disord Drug Targets 13(8):1378–1396 25345517 10.2174/1871527313666141023094720

[39] Aronowski J, Hall CE (2005) New horizons for primary intracerebral hemorrhage treatment: experience from preclinical studies. Neurol Res 27(3):268–279 15845210 10.1179/016164105X25225

[40] Xue M, Del Bigio MR (2000) Intracerebral injection of autologous whole blood in rats: time course of inflammation and cell death. Neurosci Lett 283(3):230–232 10754230 10.1016/s0304-3940(00)00971-x

[41] Wang J, Tsirka SE (2005) Contribution of extracellular proteolysis and microglia to intracerebral hemorrhage. Neurocrit Care 3(1):77–85 16159103 10.1385/NCC:3:1:077

[42] Wang J, Doré S (2007) Inflammation after intracerebral hemorrhage. J Cereb Blood Flow Metab 27(5):894–908 17033693 10.1038/sj.jcbfm.9600403

[43] Lin Q, Li M, Fang D, Fang J, Su SB (2011) The essential roles of Toll-like receptor signaling pathways in sterile inflammatory diseases. Int Immunopharmacol 11(10):1422–1432 21600309 10.1016/j.intimp.2011.04.026

[44] Hanamsagar R, Hanke ML, Kielian T (2012) Toll-like receptor (TLR) and inflammasome actions in the central nervous system. Trends Immunol 33(7):333–342 22521509 10.1016/j.it.2012.03.001 PMC3383346

[45] Lin S, Yin Q, Zhong Q, Lv FL, Zhou Y, Li JQ et al (2012) Heme activates TLR4-mediated inflammatory injury via MyD88/TRIF signaling pathway in intracerebral hemorrhage. J Neuroinflammation 9:46 22394415 10.1186/1742-2094-9-46 PMC3344687

[46] Teng W, Wang L, Xue W, Guan C (2009) Activation of TLR4-mediated NFkappaB signaling in hemorrhagic brain in rats. Mediators Inflamm 2009:473276 20150961 10.1155/2009/473276 PMC2817507

[47] Ma CX, Yin WN, Cai BW, Wu J, Wang JY, He M et al (2009) Toll-like receptor 4/nuclear factor-kappa B signaling detected in brain after early subarachnoid hemorrhage. Chin Med J 122(13):1575–1581 19719951

[48] Li T, Xia M, Gao Y, Chen Y, Xu Y (2015) Human umbilical cord mesenchymal stem cells: an overview of their potential in cell-based therapy. Expert Opin Biol Ther 15(9):1293–1306 26067213 10.1517/14712598.2015.1051528

[49] Ribeiro A, Laranjeira P, Mendes S, Velada I, Leite C, Andrade P et al (2013) Mesenchymal stem cells from umbilical cord matrix, adipose tissue and bone marrow exhibit different capability to suppress peripheral blood B, natural killer and T cells. Stem Cell Res Ther 4(5):125 24406104 10.1186/scrt336 PMC3854702

[50] Shi Y, Yang Y, Guo Q, Gao Q, Ding Y, Wang H et al (2019) Exosomes derived from human umbilical cord mesenchymal stem cells promote fibroblast-to-myofibroblast differentiation in inflammatory environments and benefit cardioprotective effects. Stem Cells Dev 28(12):799–811 30896296 10.1089/scd.2018.0242

[51] Bi S, Nie Q, Wang WQ, Zhu YL, Ma XM, Wang CM et al (2018) Human umbilical cord mesenchymal stem cells therapy for insulin resistance: a novel strategy in clinical implication. Curr Stem Cell Res Ther 13(8):658–664 30095059 10.2174/1574888X13666180810154048

[52] Tanaka E, Ogawa Y, Mukai T, Sato Y, Hamazaki T, Nagamura-Inoue T et al (2018) Dose-dependent effect of intravenous administration of human umbilical cord-derived mesenchymal stem cells in neonatal stroke mice. Front Neurol 9:133 29568282 10.3389/fneur.2018.00133 PMC5852073

[53] Li X, Hu YD, Guo Y, Chen Y, Guo DX, Zhou HL et al (2015) Safety and efficacy of intracoronary human umbilical cord-derived mesenchymal stem cell treatment for very old patients with coronary chronic total occlusion. Curr Pharm Des 21(11):1426–1432 25427243 10.2174/1381612821666141126100636

[54] Jiang J, Wang Y, Liu B, Chen X, Zhang S (2018) Challenges and research progress of the use of mesenchymal stem cells in the treatment of ischemic stroke. Brain Dev 40(7):612–626 29661589 10.1016/j.braindev.2018.03.015

[55] Baghaei K, Tokhanbigli S, Asadzadeh H, Nmaki S, Reza Zali M, Hashemi SM (2019) Exosomes as a novel cell-free therapeutic approach in gastrointestinal diseases. J Cell Physiol 234(7):9910–9926 30536895 10.1002/jcp.27934

[56] Gupta D, Zickler AM, El Andaloussi S (2021) Dosing extracellular vesicles. Adv Drug Deliv Rev 178:113961 34481030 10.1016/j.addr.2021.113961

